# O_2_-inducible H_2_O_2_-forming NADPH oxidase is responsible for the hyper O_2_ sensitivity of *Bifidobacterium longum* subsp. *infantis*

**DOI:** 10.1038/s41598-018-29030-4

**Published:** 2018-07-16

**Authors:** Kunifusa Tanaka, Takumi Satoh, Jun Kitahara, Saori Uno, Izumi Nomura, Yasunobu Kano, Tohru Suzuki, Youichi Niimura, Shinji Kawasaki

**Affiliations:** 1 Department of Molecular Microbiology, Tokyo University of Agriculture, 1-1-1 Sakuragaoka, Setagaya-ku, Tokyo 156-8502 Japan; 2 Department of Bioscience, Tokyo University of Agriculture, 1-1-1 Sakuragaoka, Setagaya-ku, Tokyo 156-8502 Japan; 3 Department of Molecular Genetics, Kyoto Pharmaceutical University, 5 Nakauchi-cho, Misasagi, Yamashina-ku, Kyoto 607-8414 Japan; 4 Faculty of Applied Biological Sciences, Gifu University, 1-1 Yanagido, Gifu, 501-1193 Japan; 5 United Graduate School of Agricultural Science, Gifu University, 1-1 Yanagido, Gifu, 501-1193 Japan

## Abstract

Bifidobacteria are beneficial anaerobes, and their O_2_ sensitivity levels differ among species as a function of unknown molecular mechanisms. *Bifidobacterium longum* subspecies *infantis* (*B. infantis*), a predominant colonizer of the gastrointestinal tract of infants, showed a hyper O_2_-sensitive growth profile with accompanying a production of H_2_O_2_. In this study, we characterized an NADPH oxidase as a key enzyme responsible for this microbe’s hyper O_2_ sensitivity. A dominant active elution peak of H_2_O_2_-forming NADPH oxidase activity was detected in the first step of column chromatography, and the purified NADPH oxidase (NPOX) was identified as a homolog of nitroreductase family proteins. The introduction of the gene encoding *B. infantis* NPOX (*npoxA*) into O_2_-tolerant *Bifidobacterium minimum* made the strain O_2_ sensitive and allowed it to produce H_2_O_2_. Knockout of the *npoxA* gene in *B. infantis* decreased the production of H_2_O_2_ and mitigated its *B. infantis* hyper O_2_ sensitivity. A transcript of *B. infantis npoxA* is induced by O_2_, suggesting that the aerobic production of toxic H_2_O_2_ is functionally conserved in *B. infantis*.

## Introduction

Oxygen (O_2_) has a negative effect on the growth of anaerobes^1,2^; however, the habitats of anaerobes, such as the gastrointestinal tract, are frequently contaminated with O_2_ that has been dissolved in food and beverages. Therefore, anaerobes must possess systems to adapt to aerated environments for survival in nature^3,4^. Recent studies have identified an O_2_-inducible O_2_- and ROS-reducing enzyme complex in obligate anaerobes such as sulfate-reducing bacteria^5,6^, *Clostridium*^7,8^, and *Bacteroides*^9^. These findings indicate that these obligate anaerobes possess excellent systems to avoid oxidative damage for maintaining their obligate anaerobiosis in aerated environments.

Several gut anaerobes such as O_2_-sensitive bifidobacteria and lactic acid bacteria are known to produce H_2_O_2_, a toxic reactive oxygen species (ROS), after exposure to O_2_, which inhibits the cell growth^10–12^. These anaerobes do not harbor the above-mentioned genes encoding the ROS-reducing enzyme complex in their genomes. NAD(P)H peroxidase or alkylhydroperoxide (Ahp) reductase systems (AhpF-AhpC or thioredoxin reductase–AhpC system), which detoxify H_2_O_2_ to H_2_O using NAD(P)H in aerobes and facultatively anaerobes^13–16^, have been identified in these anaerobes^17–19^; however, exogenous H_2_O_2_ production has been detected in these anaerobes^20,21^. These results suggest that H_2_O_2_ production and decomposition are controlled *in vivo* and play a role in the biological functions of these bacteria in aerated environments. H_2_O_2_ is highly toxic for a bacterium’s own cells; thus, the reason for this H_2_O_2_ production remains unclear^22,23^.

Members of the genus *Bifidobacterium* are gram-positive, catalase-negative anaerobes that are known to be beneficial to human health^24–26^. The degree of O_2_ sensitivity differs among species and strains in the genus^11,27–35^. de Vries and Stouthamer (1969) proposed that O_2_-sensitive *Bifidobacterium* species produce H_2_O_2_ under aerobic growth conditions through a reaction catalyzed by NADH oxidase activity, which can be detected in cell extracts^11^. According to our studies using liquid shaking cultures at different O_2_ concentrations, bifidobacterial strains can be classified into four groups: O_2_ hypersensitive (5% O_2_-sensitive), O_2_-sensitive (10% O_2_-sensitive), O_2_-tolerant (21% O_2_-tolerant), and O_2_-hypertolerant (microaerophilic)^33,36,37^. H_2_O_2_ production has been detected in strains belonging to the O_2_-hypersensitive and O_2_-sensitive groups when cell growth was inhibited by O_2_^33^. The majority of O_2_-tolerant and hyper O_2_-tolerant strains have been isolated from non-human sources such as a honeybee hindgut^38^ and bovine rumen^39^. *Bifidobacterium asteroides* (*B. asteroides*) was isolated from a honeybee hindgut and possesses a heme-catalase, which is a rare characteristic among bifidobacteria^24,38,40,41^. Several reports have revealed that the addition of catalase to the culture medium^11,33^ or the introduction of ROS-detoxifying enzymes into O_2_-sensitive *Bifidobacterium* strains^42,43^ improved their aerobic growth, indicating that H_2_O_2_ production strongly correlates with the inhibition of aerobic growth. With respect to the enzymes that produce H_2_O_2_ in bifidobacteria, an H_2_O_2_-forming NADH oxidase was identified in an O_2_-sensitive strain of *Bifidobacterium bifidum* (*B. bifidum*), a type species in the genus *Bifidobacterium*, and the purified enzyme was identified as a *b*-type dihydroorotate dehydrogenase (DHOD), which is the enzyme that catalyzes oxidation of dihydroorotate to orotate in pyrimidine biosynthesis^44^. This enzyme is suggested to be involved in H_2_O_2_ production in *B. bifidum*, but its function has not been confirmed *in vivo* because of the lack of a gene disruption technique for this bacterium.

In the present study, *Bifidobacterium longum* subspecies *infantis* (*B. infantis*) showed a hyper O_2_-sensitive growth profiles. *B. infantis* has been reported as a champion colonizer in the gut microbiota of human infants^45^ and is reported to confer benefits to premature infants in terms of promoting anti-inflammatory activity and decreasing the risk of necrotizing enterocolitis^46,47^. Use of *B. infantis* as a bioactive probiotic for infants is highly anticipated; therefore, we set out to identify the mechanisms underlying its hyper-O_2_ sensitivity. Here, we identified an enzyme that produces H_2_O_2_ in *B. infantis*. The role of this protein and the mechanisms and evolutionary significance of H_2_O_2_ production in this bacterium are discussed.

## Results

### *B. infantis* showed a hyper O_2_-sensitive growth profile

**Figure 1:**
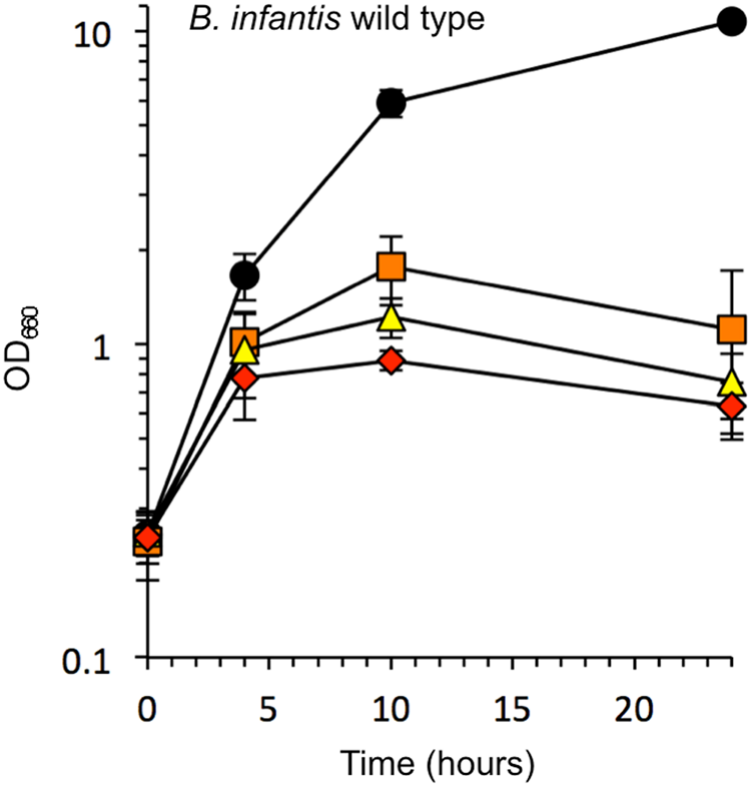
Growth of *Bifidobacterium infantis* JCM 1222^T^ in liquid shake culture at various O_2_ concentrations. Circles, 100% N_2_ cultures; squares, 5% O_2_/95% N_2_ cultures; triangles, 10% O_2_/90% N_2_ cultures; diamonds, 20% O_2_/80% N_2_ cultures. Data represent the average ± SD of three independent cultures.

The degree of O_2_ sensitivity of *B. infantis* JCM1222^T^ (=ATCC15697 = DSM20088) was tested at several O_2_ concentrations in liquid shaking cultures. *B. infantis* grew well under anoxic conditions, and the optical density at 660 nm (OD_660_) reached 8 to 10 in the final growth stage. This level of growth was one of the most productive observed in MRS medium among the tested *Bifidobacterium* species^36,37^. Although several O_2_-sensitive bifidobacterial strains, including *B. bifidum* and *Bifidobacterium longum* (*B. longum*), grew well in liquid shaking cultures at 5% O_2_^33^, the growth of *B. infantis* was found to be significantly inhibited at 5% O_2_ (Fig. 1). Therefore, we classified *B. infantis* as O_2_-hypersensitive. H_2_O_2_ production in the culture medium was detected at 10% and 20% O_2_ (Table 1).

**Table 1:** H_2_O_2_ accumulation of *B. infantis* and *B. minimum* under 0% to 20% O_2_ conditions.

O_2_ (%)	H_2_O_2_ (µM)^*a*^			
	*B. infantis* wild type	*B. infantis ∆npoxA*	*B. minimum* pkkt427^*b*^	*B. minimum* + *npoxA* in pKKT427^*c*^
0	ND^*d*^	ND	ND	ND
5	ND	ND	ND	ND
10	29 ± 10	(3 ± 1)^*e*^	ND	172 ± 13
20	31 ± 6	14 ± 10	ND	169 ± 18

^*a*^H_2_O_2_ accumulation in the medium (24 h).

^*b*^Vector pKKT427 was transformed into *B. minimum*.

^*c*^The *B. infantis npoxA* gene was cloned in pKKT427 and transformed into *B. minimum*.

^*d*^ND, not detected (less than 3 µM).

^*e*^Data in parentheses are close to the reliable detection limit.

Data represent the average ± SD of three independent cultures.

### Elution profiles of NAD(P)H oxidase activity in the first step of column chromatography

**Figure 2:**
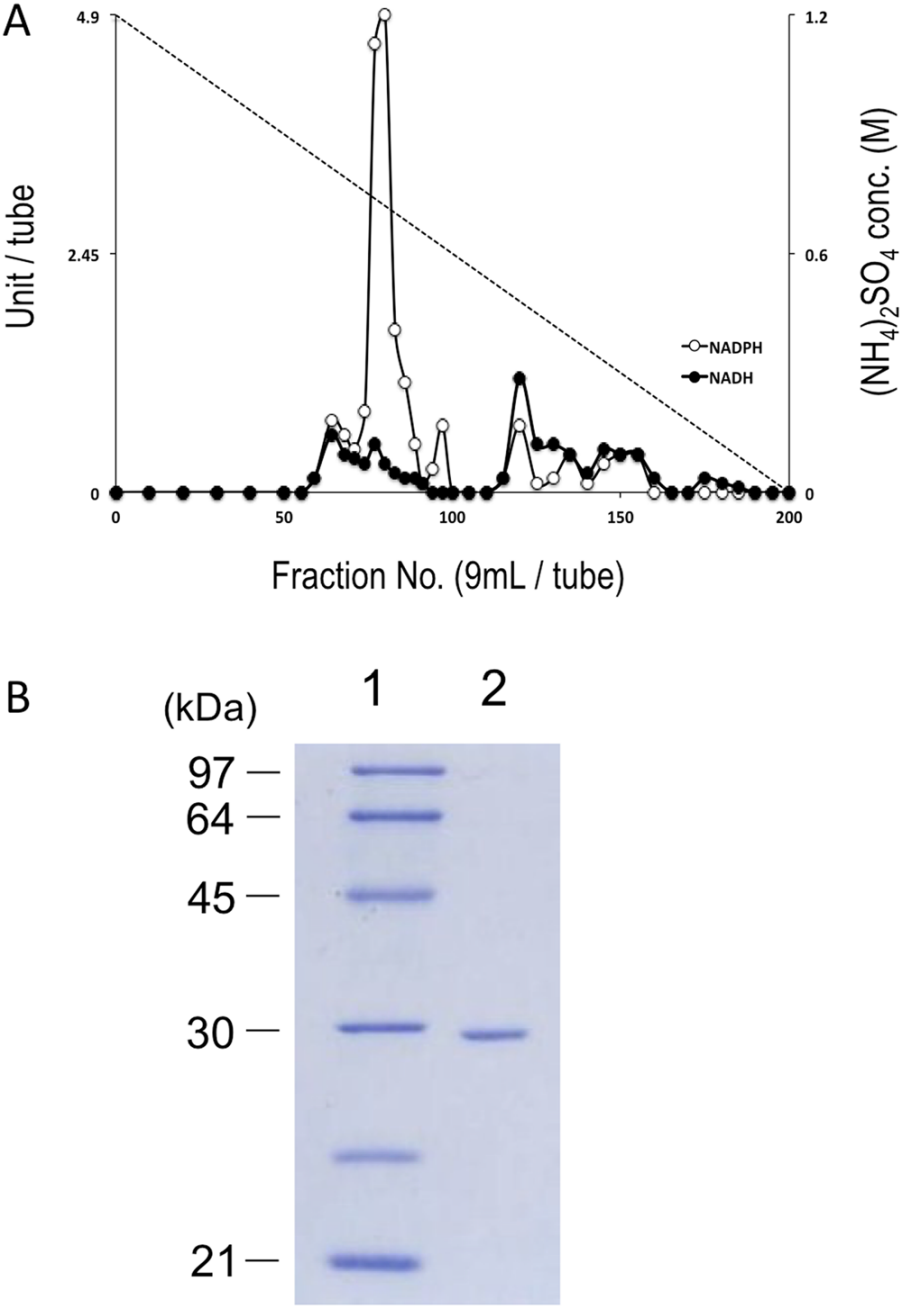
(**A**) Chromatographic elution profiles of *B. infantis* NADH and NADPH oxidases. Cell extracts, after treatment with streptomycin sulfate and ammonium sulfate, were applied to a Butyl-TOYOPEARL column equilibrated with 1.2 M ammonium sulfate in 50 mM potassium phosphate buffer (pH 7.0). After sample loading, the bound proteins were eluted with a linear gradient of ammonium sulfate, from 1.2 to 0 M, dissolved in the same buffer. Black circles, NADH oxidase activity; white circles, NADPH oxidase activity; dashed line, ammonium sulfate concentration (conc.). (**B**) SDS-PAGE of the purified *B. infantis* NPOX protein. After electrophoresis, the gel was stained with Coomassie brilliant blue. The protein standards (lane 1) and purified protein (lane 2) are indicated, along with the corresponding molecular masses (indicated on the left in kDa).

We previously identified the enzyme fractions that are involved in H_2_O_2_ production in an O_2_-sensitive *B. bifidum* strain using a Butyl-TOYOPEARL column^44^. In this study, we performed the same experiments for *B. infantis*. Proteins in the cell free extracts from *B. infantis* were fractionated according to their hydrophobic properties using a Butyl-TOYOPEARL column (Fig. 2A). One dominant elution peak of NADPH-dependent oxidase activity was detected, whereas NADH-dependent oxidase activity was detected as a minor elution peak. These major and minor peak fractions showed an H_2_O_2_-forming oxidase reaction in which stoichiometric production of H_2_O_2_ was detected by means of the reduction of O_2_. No significant NAD(P)H oxidase activity was detected in either the unbound fraction or the washed fractions in the first step of column chromatography.

### Purification and characterization of the NADPH oxidase in *B. infantis*

The fractions that showed NADPH oxidase activity were collected and purified by additional column chromatography steps. At each purification step, only one predominant active elution peak was detected. After the final step of affinity chromatography, the purified fractions appeared as a single band (29 kDa) by SDS-PAGE (Fig. 2B). The N-terminal sequence of the purified NADPH oxidase (NPOX) was sequenced, and the amino acid sequence (MVTNATIEALLGRRSIRKFK) showed 100% identity with the N-terminal sequence of the Blon_2447 gene product of *B. infantis* ATCC15697 in the genome database.

The gene encoding NPOX (designated as *npoxA*) was cloned from the *B. infantis* genome, and the amino acid sequence identity (% amino acid identity calculated from ClustalW alignment) showed 100% to that derived from BLON_RS12650 (Blon_2447). The translated product of *B. infantis npoxA* showed homology to proteins of the bacterial nitroreductase family and encodes a protein of 254 amino acids that shows ~35% identity to *Escherichia coli* oxygen-insensitive NADPH nitroreductase NfsA (accession number P17117)^48^, *Bacillus subtilis* NADPH-dependent nitroreductase NfrA1 (32% identity, accession number P39605)^49^, and *Vibrio harveyi* NADPH-flavin oxidoreductase FRP1 (35% identity, accession number Q56691)^50^. *B. subtilis* NfrA1 is reported to catalyze H_2_O_2_-forming NADH oxidase activity^51^, and *V. harveyi* FRP1 is reported to catalyze an FMN reductase reaction that is involved in a luciferase reaction that generates light in luminescent bacteria^52^. These NPOX homologs utilize NADH or NADPH or both as an electron donor to reduce substrates. The protein homologs are widely distributed in the genus *Bifidobacterium*. Homologous proteins were found in several *B. longum* strains (99% to 100%), *Bifidobacterium breve* (85% identity), *B. bifidum* (70% identity), *Bifidobacterium minimum* (*B. minimum*) (58% identity), and *B. asteroides* (52% identity). None of these proteins have been characterized in terms of function.

### Enzymatic properties

The purified enzyme showed high affinity for NADPH, rather than NADH, as the electron donor. The *K*_*m*_ values for NADPH and NADH when O_2_ was used as an electron acceptor were 1.38 ± 0.12 and 113.3 ± 7.3 µM, respectively. The purified *B. infantis* NPOX showed a typical flavoprotein spectrum with absorbance maxima at 280, 373, and 444 nm (Fig. S2). The bound flavin was identified as FMN by HPLC analysis. In the NADPH oxidase reaction, stoichiometric production of H_2_O_2_ was detected following the reaction with O_2_; e.g., 1 µg enzyme consumed 28.3 nmol O_2_/5 min/mL, and 13.8 nmol O_2_/mL was produced after the addition of catalase. This result indicated that the purified enzyme reduces O_2_ by two reducing equivalents to H_2_O_2_. The purified enzyme used flavins and nitro compounds as electron acceptors under anoxic conditions (Table 2). The pH optimum for the NADPH oxidase reaction was found to be 5.5–6.0. The temperature optimum was approximately 30–40 °C, and activity significantly decreased at temperatures above 40 °C. The specific activity of this protein under air-saturated conditions at 37 °C was 13.8 ± 4.3 U/mg protein.

**Table 2:** Identification of electron acceptors of *B. infantis* NPOX.

Electron acceptor^*a*^	Relative activity (%)^*b*^
O_2_	100
DCIP	358
FAD	229
FMN	74
Nitrobenzene	2090
Nitrofurazone	725

^*a*^Relative activity (%) was calculated in comparison with the activity of NADPH oxidase.

Data are the average of two independent measurements that varied by less than 5%.

### Transformation of the *B. infantis npoxA* into an O_2_-tolerant *B. minimum* strain

**Figure 3:**
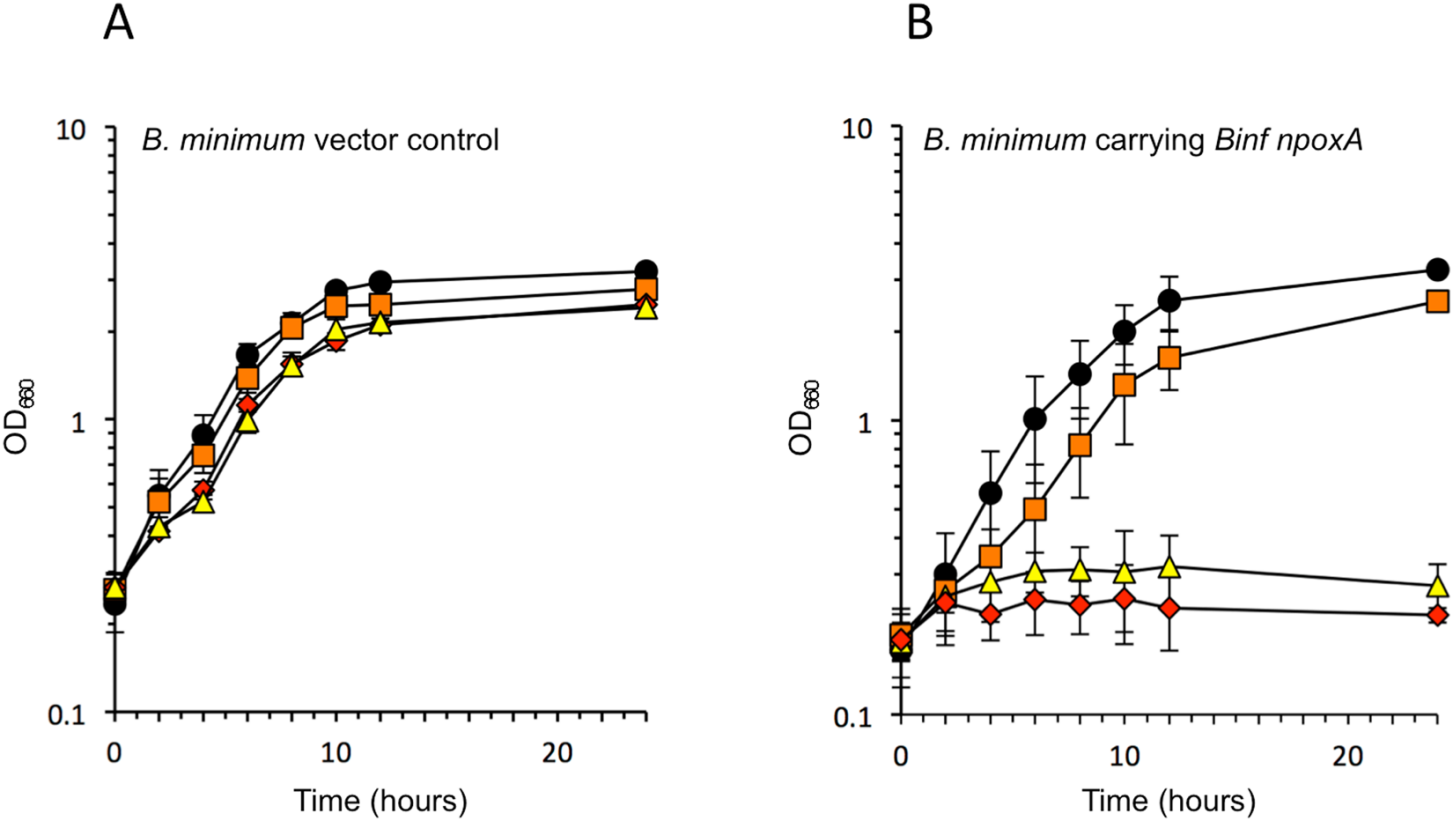
Growth of *Bifidobacterium minimum* DSM 20102^T^ in liquid shake culture at various O_2_ concentrations with (**B**) or without (**A**) the *B. infantis npoxA* gene in the vector pKKT427. Circles, 100% N_2_ cultures; squares, 5% O_2_/95% N_2_ cultures; triangles, 10% O_2_/90% N_2_ cultures; diamonds, 20% O_2_/80% N_2_ cultures. Data represent the average ± SD of three independent cultures.

*B. minimum* DSM 20102^T^, which is an isolate from sewage^53^, showed an O_2_-tolerant profile^32,37^, and liquid shaking growth was not significantly inhibited in the presence of 20% O_2_ (Fig. 3A). Therefore, we classified *B. minimum* as a member of the O_2_-tolerant group and used this microbe for further study. The *E. coli-Bifidobacterium* shuttle vector pKKT427, originally developed for the transformation of *B. longum* strain 105-A^54,55^, was successfully transformed into *B. minimum* in this study; therefore, we used this vector to express *B. infantis* NPOX in *B. minimum*. *B. infantis npoxA* was cloned into pKKT427, and then the growth of transformants was tested at different O_2_ concentrations. The production of H_2_O_2_ was not detected in a *B. minimum* wild-type strain carrying pKKT427 in the presence of O_2_ (Table 1). However, *B. minimum* carrying *npoxA* in pKKT427 showed an O_2_-sensitive growth profile in the presence of 10% and 20% O_2_ with accompanying the production of H_2_O_2_ (Fig. 3B, Table 1). These results indicated that *B. infantis* NPOX is responsible for the O_2_ sensitivity of and H_2_O_2_ production by *B. minimum*.

### The growth of a *B. infantis npoxA* gene knockout strain

**Figure 4:**
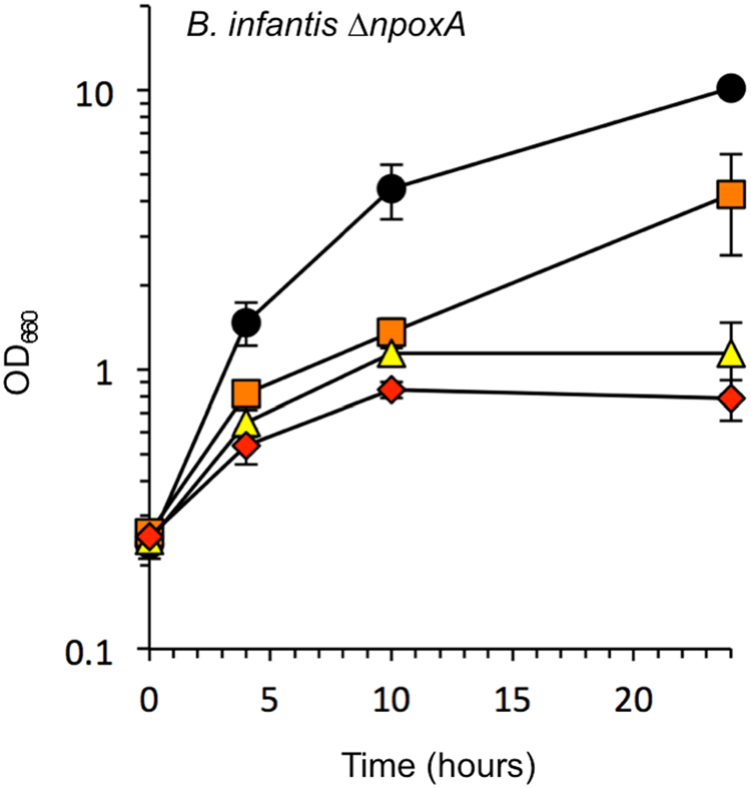
Growth of the *∆npoxA* mutant of *B. infantis* in liquid shake culture at various O_2_ concentrations. Circles, 100% N_2_ cultures; squares, 5% O_2_/95% N_2_ cultures; triangles, 10% O_2_/90% N_2_ cultures; diamonds, 20% O_2_/80% N_2_ cultures. Data represent the average ± SD of four independent experiments.

Single-gene transformation of the O_2_-tolerant *B. minimum* strain significantly increased H_2_O_2_ production and O_2_ sensitivity. To determine the function of the *npoxA* product in *B. infantis*, we constructed an *npoxA* deletion mutant (*∆npoxA*) (Fig. S1), and then the growth of this mutant was compared with that of a wild-type strain of *B. infantis*. *B. infantis ∆npoxA* grew well at 5% O_2_, and H_2_O_2_ production was significantly decreased at 10% and 20% O_2_ (Fig. 4, Table 1). These results indicated that the *npoxA* product contributes to both aerobic growth inhibition and H_2_O_2_ production in *B. infantis*. Candidates for the production of residual H_2_O_2_ were the minor active fractions of the NADH-dependent H_2_O_2_-forming oxidase (Fig. 2A).

### Expression profiles of genes encoding *B. infantis* NADPH oxidase and alkylhydroperoxide reductase

**Figure 5:**
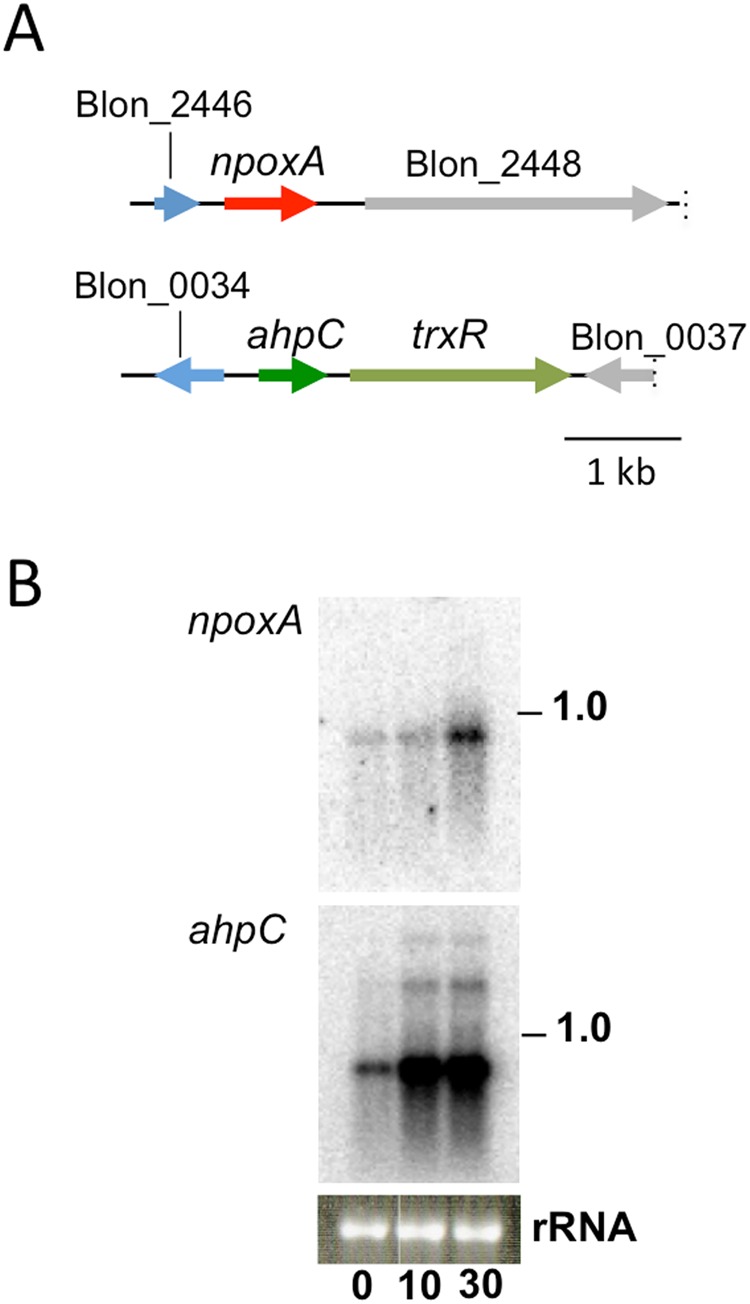
Genome structures and gene expression profiles of *B. infantis npoxA* and *ahpC*. (**A**) Genome structure of the *npoxA* (upper) and the *ahpC* (lower). (**B**) Northern blot analyses of 5 µg of *B. infantis* total RNA probed with *npoxA* or *ahpC*. 0, immediately before the start of aeration with 5% O_2_ in the mid-exponential phase; 10, after 10 min; 30, after 30 min. The estimated sizes of the observed transcripts are indicated on the right. Ethidium bromide staining of the ribosomal RNA (rRNA) to confirm equal RNA loading is shown below the autoradiogram. The membranes were reused for reprobing different probes.

To determine the effect of O_2_ on the expression of the gene encoding *B. infantis* NADPH oxidase (*npoxA*), we performed a Northern blot analysis. The result showed that *B. infantis npoxA* was significantly upregulated within 30 min after the start of 5% O_2_ aeration (Fig. 5B). We also determined the expression of genes encoding alkylhydroperoxide reductase (Ahp), which is an enzyme complex composed of AhpF and AhpC that detoxifies ROS. TrxR is an AhpF homolog in *B. infantis*, and *ahpC-trxR* genes are tandemly located in the genome (Fig. 5A). Northern analyses indicated that *B. infantis ahpC* was strongly upregulated within 10 minutes after the start of aeration (Fig. 5B). These results indicate that *B. infantis* express the genes for ROS production and for ROS detoxification in response to O_2_ stress.

## Discussion

The growth of bifidobacterial strains is inhibited by O_2_, and this inhibition has been shown to occur in conjunction with H_2_O_2_ production. To date, *b*-type DHOD from an O_2_-sensitive strain of *B. bifidum*, has been identified as the only purified H_2_O_2_-forming NADH oxidase in the genus *Bifidobacterium*. In this study, we detected a dominant H_2_O_2_-forming NADPH oxidase activity peak in an O_2_-hypersensitive strain of *B. infantis*. This NADPH-specific peak was not detected in *B. bifidum* using the same hydrophobic column chromatography^44^, thus suggesting that the mechanisms of O_2_ sensitivity in these O_2_-sensitive strains differ from each other.

Purified *B. infantis* H_2_O_2_-forming NPOX showed high similarity (~30% identity) with proteins of the bacterial nitroreductase family. *B. infantis* NPOX exhibited nitroreductase- and flavin reductase-activity under anoxic conditions. The role of *B. infantis* H_2_O_2_-forming NPOX under anaerobic conditions is unclear; however, based on the O_2_-inducible gene expression profile, NPOX is expected to be functionally involved in the aerobic life of *B. infantis*. Similar expression of a transcript, from a gene that encodes an NPOX ortholog (Gene ID: BL0139), was observed by Oberg *et al*. in microarray data from the H_2_O_2_-sensitive strain *B. longum* D2975 and the H_2_O_2_-tolerant strain *B. longum* NCC2705^56^. The NPOX homolog transcript was induced only in the H_2_O_2_-sensitive D2975 strain under oxidative stress. To clarify the expression of NPOX and its correlation with bifidobacterial O_2_ sensitivity, it would be desirable to compare the expression profiles and kinetics of NPOX homologs in other O_2_-tolerant and O_2_-sensitive strains^37^.

H_2_O_2_ is highly toxic to cells, and therefore, *Bifidobacterium* strains need to conserve systems to reduce the amount of H_2_O_2_ to avoid cell death. Alkylhydroperoxide reductase, a component of the AhpF-AhpC system that is known to decompose H_2_O_2_ efficiently, is widely conserved in bifidobacterial genomes. Although the function of this enzyme complex has not been characterized in the genus *Bifidobacterium*, the upregulation of *ahpC* (or the protein AhpC) in response to O_2_ or H_2_O_2_ stress has been reported in strains such as *Bifidobacterium animalis* subsp. *lactis*^35^ and *B. longum*^56^. These data suggested that *ahpC* is involved in the oxidative stress protection of bifidobacterial strains. In the present study, *B. infantis* induced *ahpC* within 10 minutes after exposure to O_2_. Interestingly, the induction of *ahpC* was more rapid than that of *B. infantis npoxA* (Fig. 5). This gene expression program is considered to be important for the strain to rapidly prepare the defense system against the generation of H_2_O_2_.

In this study, knockout of *npoxA* in *B. infantis* enabled the strain to grow under microaerobic conditions, with a decrease in H_2_O_2_ production under aerobic conditions. Improvement of bacterial O_2_ sensitivity by single-gene knockout has been demonstrated in the obligate anaerobe *Bacteroides fragilis*, with deletion of *oxe* enabling the strain to grow under micro-oxic conditions (as high as 2% O_2_)^57^. Although the function of the *oxe* gene product has not yet been identified, a mutation in *oxe* enhanced the H_2_O_2_-scavenging activity of the mutant relative to that of the wild type. The reason for the production of a toxic H_2_O_2_ in anaerobes has been unknown; however, it is obvious that both *B. fragilis* and *B. infantis* control the production and degradation of H_2_O_2_ according to their own genetic programs.

We conclude here that *B. infantis* induces the expression of NPOX in response to an increase in O_2_, leading to the production of H_2_O_2_ to maintain anaerobiosis. *B. infantis ∆npoxA* still produced H_2_O_2_ at elevated O_2_ concentrations, indicating the presence of another H_2_O_2_-forming oxidase *in vivo*. A *b*-type DHOD, that is conserved in the genome of *B. infantis*, is a likely candidate. Further characterization of the residual H_2_O_2_ production in *B. infantis*, as well as analysis of the enzyme kinetics of the O_2_-tolerant *B. minimum* NPOX homolog, will be needed to describe the mechanisms of anaerobiosis in these strains.

## Methods

### Bacterial strain and growth conditions

*B. infantis* (*Bifidobacterium longum* subsp. *infantis*) JCM 1222^T^ (=ATCC 15697^T^, DSM 20088^T^) was used in this study. *B. infantis* was grown anaerobically at 37 °C in modified MRS medium (pH 6.7). The culturing conditions under several O_2_ concentrations were as described previously^33^.

### Chemicals

All chemicals were of analytical grade. ß-NADPH, ß-NADH, FAD, FMN, riboflavin, nitrobenzene, nitrofurazone, and 2,6-dichlorobenzenoneindophenol (DCIP) were from Sigma. Water was prepared with an Ellix-10 Milli-Q ultra-pure water system (Millipore, Tokyo, Japan).

### Enzyme purification

Microaerobically grown *B. infantis* cells (cultured statically but stirred with a magnetic stirrer in 3 liters of medium in a 5-liter flask with a cotton plug) were harvested for enzyme purification. NAD(P)H oxidase activity was assayed spectrophotometrically in 1 ml of air saturated 50 mM potassium phosphate buffer (pH 6.5) at 37 °C. The reaction was started by the addition of enzyme solution, and the decrease in absorbance at 340 nm (ε_340_ = 6,220 M^−1^·cm^−1^) was monitored with a spectrophotometer (HITACHI U-3300, Hitachi, Japan). One unit of activity was defined as the amount of enzyme that catalyzes the oxidation of 1 µmol NAD(P)H per minute.

Microaerobically grown *B. infantis* cells (80 g) were suspended in 240 ml of 50 mM potassium phosphate buffer, pH 6.5, containing 0.1 mM DTT, 0.2 mM PMSF, and 5 mM EDTA, and then the cells were disrupted by treatment with a French pressure cell at 140 MPa. All purification procedures were carried out at 4 °C or on ice. Cell free extracts (CFE) were obtained by removing cell debris by centrifugation at 39,000 *g* for 15 min. The cytoplasmic solution obtained by ultracentrifugation at 100,000 *g* for 2 h was treated with 10% streptomycin sulfate (dissolved in 10 mM potassium phosphate buffer, pH 7.0) to remove nucleic acids. After centrifugation at 39,000 *g* for 15 min, the supernatant was fractionated by the stepwise addition of solid ammonium sulfate (20%). The supernatant obtained after centrifugation at 30,000 g was dissolved in 50 mM potassium phosphate buffer, pH 7.0, containing 1 M ammonium sulfate, 5 mM EDTA, 0.2 mM phenylmethanesulfonyl fluoride (PMSF), and 0.1 mM dithiothreitol (DTT). After centrifugation at 39.000 *g* for 15 min, the supernatant was applied on a hydrophobic interaction chromatography using Butyl-TOYOPEARL 650 s (TOSOH, Tokyo, Japan) column chromatography. The enzyme was eluted with a linear gradient from 1.2 M to 0 M ammonium sulfate dissolved in the same buffer. After the Butyl-TOYOPEARL chromatography, active fractions of NADPH oxidase activity were obtained. The solution after Butyl-TOYOPEARL chromatography was dialyzed against 50 mM potassium phosphate buffer, pH 6.5, containing 0.5 mM EDTA, 0.2 mM PMSF, and 0.1 mM DTT for 6 h, and this procedure was repeated 3 times using freshly prepared buffer each time. The enzyme solution was then applied to DEAE Sephacel (GE Healthcare, USA) column chromatography. The column was sequentially washed with the same buffer containing 0 mM and 150 mM NaCl, and eluted with buffer containing 250 mM NaCl. The active fractions were pooled and dialyzed for 12 h against 10 mM potassium phosphate buffer, pH 6.5, containing 0.5 mM EDTA and 0.1 mM DTT and subjected to hydroxyapatite (WAKO, Japan) column chromatography. After applying the enzyme solution to the hydroxyapatite column, the protein was sequentially washed with 10 mM and 50 mM potassium phosphate buffer, pH 6.5, and eluted with 100 mM potassium phosphate buffer, pH 6.5. The active fractions were collected and concentrated using Amicon Ultra centrifugal filter units (30,000 Da cutoff; MILLIPORE, Cork, Ireland). The concentrated enzyme was applied to POLOS HQ/H (PerSeptive Biosystems, Framingham, USA) column chromatography. The column was sequentially washed with 50 mM potassium phosphate buffer (pH 6.5), then eluted with a linear gradient of 0 mM–250 mM NaCl. The active fraction was collected and concentrated using Amicon Ultra. After this chromatography, the enzyme purity in the active fractions was checked by SDS-PAGE with Coomassie Brilliant Blue G-250 staining or silver staining. By using this enzyme concentrator, the basal buffer of the enzyme solution was changed to 50 mM potassium phosphate buffer, pH 6.5, by diluting the concentrated enzyme solution 100-fold with the new buffer, concentrating to the original volume, and then repeating the dilution and concentration steps; the second concentrated enzyme solution was subjected to enzyme assay. Protein concentration was determined by the dye-binding assay^58^.

The purified enzyme was subjected to SDS-PAGE and electroblotted onto polyvinylidene difluoride membranes (NIPPON GENETICS, Tokyo, Japan). The N-terminal amino acid sequence was determined by the Edman degradation method using the peptide sequencer described previously^7^. The UV-Vis absorption spectrum was recorded on a Hitachi U3300 spectrophotometer (Hitachi, Tokyo, Japan) using a 1 cm path length quartz cuvette.

The bound flavins were extracted and determined by HPLC analysis with fluorescence detector according to a previously described method using a CAPCELL PACK C18 column (4.6 mm I.D. × 150 mm L; SHISEIDO CO., LTD. Tokyo, Japan) with 5 mM ammonium acetate in methanol as the mobile phase^7^. Riboflavin, FAD and FMN were used as standards.

### Enzyme properties

The optimal pH of the purified enzyme was determined in 50 mM potassium phosphate buffer in the pH range of pH 5.0 – pH 8.0 at 37 °C, which is the optimum growth temperature for *B. infantis*. The temperature optimum of the purified enzyme was determined in 50 mM potassium phosphate buffer, pH 6.5, over a temperature range from 25 to 60 °C.

H_2_O_2_ production by the *B. infantis* NAD(P)H oxidase reaction was detected while monitoring O_2_ production using an oxygen electrode by adding catalase into the reaction vessel^8,33,44^. Catalase catalyzes the stoichiometric conversion of 1 mol H_2_O_2_ to 1 mol H_2_O and 1/2 mol O_2_. The H_2_O_2_-forming type NAD(P)H oxidase produces 50% O_2_ after the addition of catalase to the total amount of O_2_ consumed by the NAD(P)H oxidase reaction.

The substrate specificity of the purified enzyme was assayed spectrophotometrically at 37 °C using 50 mM potassium phosphate buffer, pH 6.5. Electron acceptors for a purified enzyme were investigated by adding various substrates (final concentration, 50 µM) to the anaerobic cuvette together with the enzyme solution. For all acceptors except O_2_, the reactions were carried out under anoxic conditions by purging the anaerobic glass cuvette containing the reaction buffer with O_2_-free argon gas. NAD(P)H:O_2_ oxidoreductase activity was assayed under air saturated conditioin. NAD(P)H (final 150 µM) was used as an electron donor for the enzyme. For the reactions of NAD(P)H:acceptor oxidoreductase, one unit of activity was defined as the amount of enzyme that catalyzes the reduction of 1 µmol of NAD(P)H (ε_340_ = 6,220 M^−1^ cm^−1^) per minute. For the assay of DCIP, one unit of activity was defined as the amount of enzyme that catalyzes the reduction of 1 µmol of DCIP (ε_600_ = 22,000 M^−1^·cm^−1^) per minute. The values for relative activities (%) and the specific activities (U/mg protein) are the average of two independent measurements that varied by less than 5%. The apparent *K*_*m*_ values for NADH and NADPH were determined by varying the concentrations of both NAD(P)H in 50 mM potassium phosphate buffer, pH 6.5 using the Enzyme Kinetics Module 1.3 (SigmaPlot 11, SYSTAT Software, Chicago, IL). Initial rates were determined from linear plots of NAD(P)H reduction.

### Northern hybridization

Northern hybridization was performed as described previously^8^. Total RNA (5 µg) extracted from *B. infantis* cells harvested before or after the 5% O_2_ stress was loaded on agarose gels and blotted onto nylon membranes (Hybond N^+^, GE Healthcare, Cicago, IL, USA). The membrane was then probed with the ^32^P-labeled gene probe of *B. infantis npoxA*. The membrane was subsequently stripped of the probe and reprobed with a probe of *B. infantis ahpC*. Northern hybridization using the same membrane was repeated twice to confirm the results. The following oligonucleotide primer pairs were used to amplify the probes for *B. infantis npoxA*: 5′-ATGGTTACCAACGCAACAAT and 52-CTAATCCAGACGGAAGCCCT, *B. infantis ahpC*: 5′-ATGACTCTTCTGCAGCATGA and 52-TCACAGCTGGCCGACGAGGT. Before hybridization with a different probe, the membrane was washed at 90 °C for 2 minutes in a stripping buffer (5% SDS in 50 mM Tris-HCl, pH7.5) and reused for the next northern analysis by reprobing with a different gene probe.

### Expression of *B. infantisnpoxA* in *B. minimum*

*B. infantis npoxA* including its promoter region was amplified by PCR using the forward primer (5′-TAAAAGCTTTGGATGATGGTTTCTGTTGG) included a *Hind*III restriction site, and the reverse primer (5′-TAAGCGGCCGCCTAATCCAGACGGAAGCCCTG) included a *Not*I restriction site. The purified PCR product was digested with *Hind*III and *Not*I and ligated into *Hind*III- and *Not*I-digested pKKT427 vector^54^, resulting in the plasmid pBInpoxA, which was transformed into *B. minimum* by electroporation. *B. minimum* cells carrying pBInpoxA or cells carrying a control vector pKKT427 were tested the growth in MRS medium containing spectinomycin (Sp) (80 µg/ml) under several O_2_ concentrations.

### Gene knockout of *npoxA*

A plasmid pKO403Cm-*∆npoxA* for the knockout of the *B. infantis npoxA* gene was constructed as follows (Fig. S1). The PCR primers were designed according to the *npoxA* gene sequence (BLON_RS12650) and the flanking genomic regions of *B. infantis* JCM1222^T^ (=ATCC15697) (GenBank Accession no. NC_011593). To obtain the *npoxA* deletion DNA fragments, upstream (1.35 kb) and downstream (1.36 kb) regions of the putative *npoxA* gene of *B. infantis* were amplified by PCR using the primers shown in Fig. S1B. The produced DNA fragments were connected to the upstream and downstream regions of the Sp resistance gene (*Sp*^r^) by Golden Gate Assembly^59^ with *Sap*I as the Type IIS restriction enzyme. The obtained fragments were cloned into pKO403Cm, a *Bifidobacterium*-*Escherichia coli* shuttle vector^60^, which carries a Golden Gate cloning site, chloramphenicol (Cm) resistance gene (*Cm*^r^), and temperature-sensitive replicon origin^60^. *B. infantis* cells were transformed with pKO403Cm-*∆npoxA* by electroporation^55^. After transformation, the cells were spread and cultured at 42 °C on MRS plates containing Sp (50 µg/ml). The transformants were transferred onto two MRS agar plates containing either Sp or Cm. The obtained Sp-resistant/Cm-sensitive transformants were selected as candidates for the double-crossover mutant. The deletion of *npoxA* was confirmed by PCR using Primer-1 and Primer-2 (Fig. S1C) and nucleotide sequencing to confirm the precise disruption.

## Electronic supplementary material

Supplementary Information FigS1-S3
