# Supplementary material for: O2-inducible H2O2-forming NADPH oxidase is responsible for the hyper O2 sensitivity of Bifidobacterium longum subsp. infantis
Source: Sci Rep. 2018 Jul 16;8:10750. doi: 10.1038/s41598-018-29030-4 (PMC6048055; doi:10.1038/s41598-018-29030-4)
Supplement: Supplementary file 1 — Supplementary Information FigS1-S3 [file 41598_2018_29030_MOESM1_ESM.pdf]

Supplemental Information

O_2-inducible H_2O_2-forming NADPH oxidase is responsible for the hyper O_2 sensitivity of *Bifidobacterium longum* subsp. *infantis*

Kunifusa Tanaka[2], Takumi Satoh[1], Jun Kitahara[2], Saori Uno[2], Izumi Nomura[4], Yasunobu Kano[3], Tohru Suzuki[4,5], Youichi Niimura[1,2], Shinji Kawasaki[1,2*]

[1]Department of Molecular Microbiology, Tokyo University of Agriculture, 1-1-1 Sakuragaoka, Setagaya-ku, Tokyo 156-8502, Japan
[2]Department of Bioscience, Tokyo University of Agriculture, 1-1-1 Sakuragaoka, Setagaya-ku, Tokyo 156-8502, Japan
[3]Department of Molecular Genetics, Kyoto Pharmaceutical University, 5 Nakauchi-cho, Misasagi, Yamashina-ku, Kyoto 607-8414, Japan.
[4]Faculty of Applied Biological Sciences, Gifu University, 1-1 Yanagido, Gifu, 501-1193 Japan.
[5]United Graduate School of Agricultural Science, Gifu University, 1-1 Yanagido, Gifu, 501-1193 Japan.

*Correspondence: Shinji Kawasaki, kawashin@nodai.ac.jp, Tel&Fax+81-3-5477-2764

A

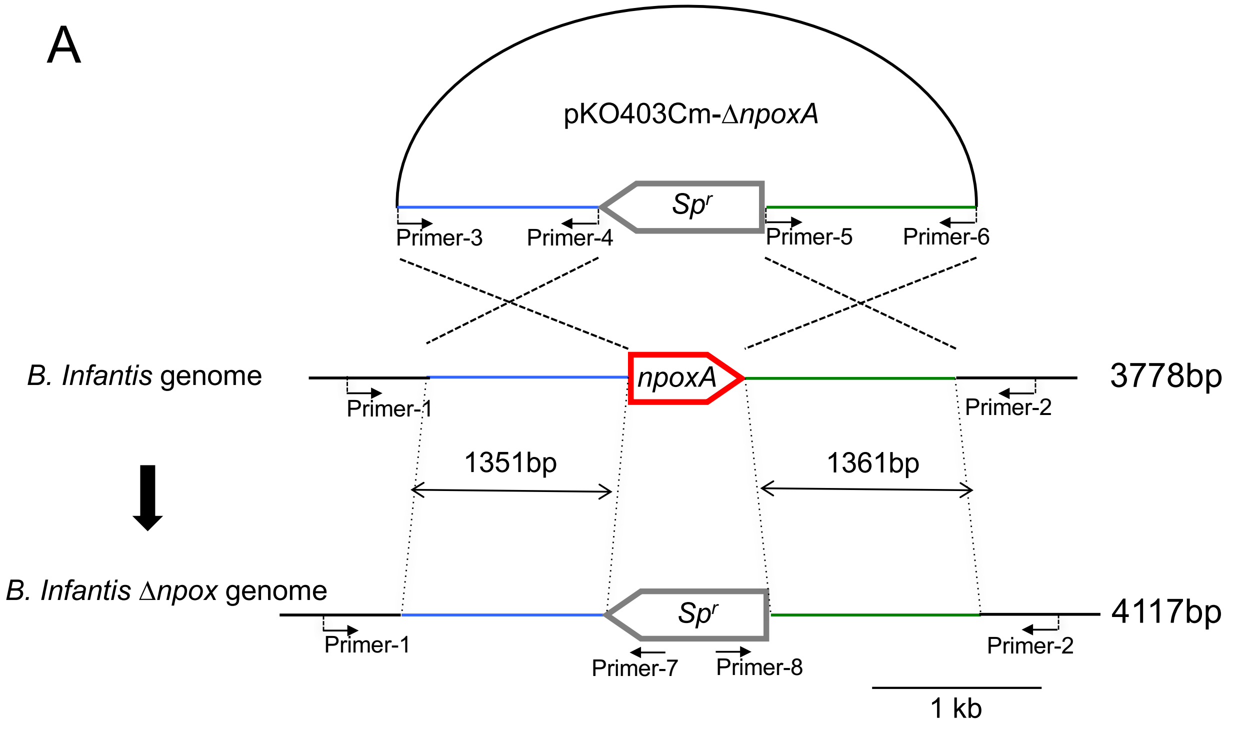

B

Primer-1	CAG GCC AGC GTA ATC ACA G
Primer-2	GTG CCG GAC AAC GAA GTC AA
Primer-3	acaGTTCGATGACGTTATCTCCCT
Primer-4	aagGGCTTTATTGTCGCACGG
Primer-5	gaaTGAGACCAGGACCAACGA
Primer-6	ctaGAGCGCATCGATTTGTTTCCA
Primer-7	AGC AGT TCG TAG TTA TCT TGG AG
Primer-8	GTT ATT TAT AGA TTT CAT TGG CTT

C

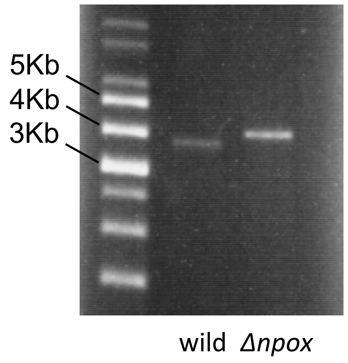

Supplementary Figure S1
Procedure for *B. infantis npox* gene disruption. (A) A plasmid pKO403Cm-Δ*npoxA* for the knockout of the *B. infantis npox* gene was constructed. (B) Primers used for the gene knockout experiments. Small letters are the 5' overhang for the Golden Gate cloning experiment. (C) The deletion of *npoxA* was confirmed by PCR using Primer-1 and Primer-2. The PCR products were sequenced to confirm the precise disruption.

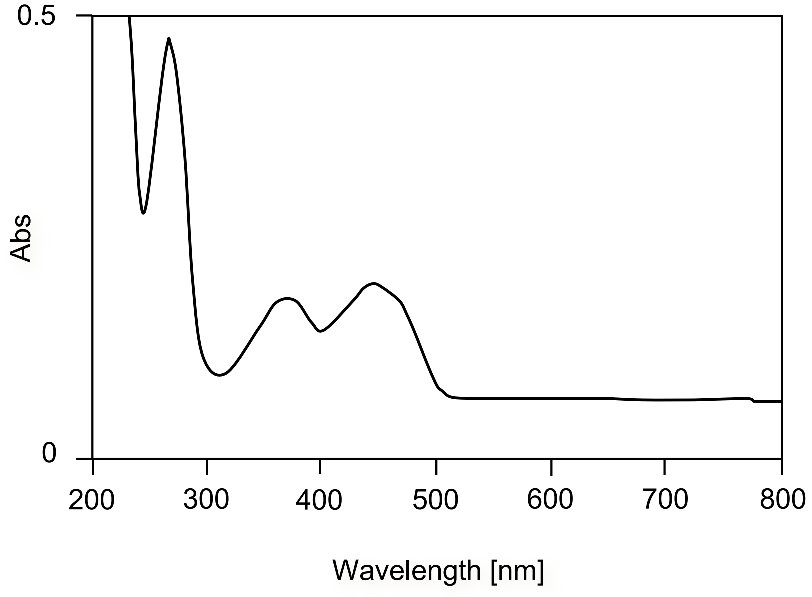

Supplementary Figure S2
Absorption spectrum of the *B. infantis* NPOX protein. The absorption (Abs) spectrum of the purified enzyme was determined in 50 mM sodium phosphate, pH 7.0.

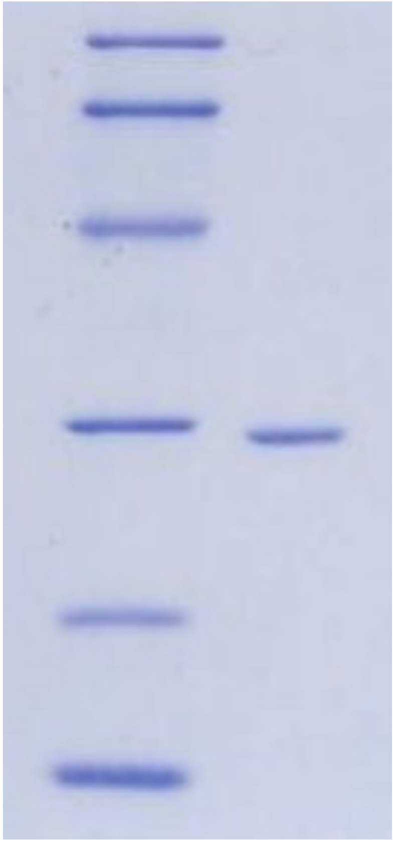

Supplementary Figure S3

Original SDS-PAGE image of the purified *B. infantis* NPOX used to generate Figure 2B.
